# Supplementary material for: At the Intersection of Cardiology and Oncology: TGFβ as a Clinically Translatable Therapy for TNBC Treatment and as a Major Regulator of Post-Chemotherapy Cardiomyopathy
Source: Cancers (Basel). 2022 Mar 19;14(6):1577. doi: 10.3390/cancers14061577 (PMC8946238; doi:10.3390/cancers14061577)

Table S1: List Cancer Types Across 32 Studies Used for Database Analysis

Combined Study

This combined study contains samples from 32 studies

Acute Myeloid Leukemia (TCGA, PanCancer Atlas)
Adrenocortical Carcinoma (TCGA, PanCancer Atlas)
Bladder Urothelial Carcinoma (TCGA, PanCancer Atlas)
Brain Lower Grade Glioma (TCGA, PanCancer Atlas)
Breast Invasive Carcinoma (TCGA, PanCancer Atlas)
Cervical Squamous Cell Carcinoma (TCGA, PanCancer Atlas)
Cholangiocarcinoma (TCGA, PanCancer Atlas)
Colorectal Adenocarcinoma (TCGA, PanCancer Atlas)
Diffuse Large B-Cell Lymphoma (TCGA, PanCancer Atlas)
Esophageal Adenocarcinoma (TCGA, PanCancer Atlas)
Glioblastoma Multiforme (TCGA, PanCancer Atlas)
Head and Neck Squamous Cell Carcinoma (TCGA, PanCancer Atlas)
Kidney Chromophobe (TCGA, PanCancer Atlas)
Kidney Renal Clear Cell Carcinoma (TCGA, PanCancer Atlas)
Kidney Renal Papillary Cell Carcinoma (TCGA, PanCancer Atlas)
Liver Hepatocellular Carcinoma (TCGA, PanCancer Atlas)
Lung Adenocarcinoma (TCGA, PanCancer Atlas)
Lung Squamous Cell Carcinoma (TCGA, PanCancer Atlas)
Mesothelioma (TCGA, PanCancer Atlas)
Ovarian Serous Cystadenocarcinoma (TCGA, PanCancer Atlas)
Pancreatic Adenocarcinoma (TCGA, PanCancer Atlas)
Pheochromocytoma and Paraganglioma (TCGA, PanCancer Atlas)
Prostate Adenocarcinoma (TCGA, PanCancer Atlas)
Sarcoma (TCGA, PanCancer Atlas)
Skin Cutaneous Melanoma (TCGA, PanCancer Atlas)
Stomach Adenocarcinoma (TCGA, PanCancer Atlas)
Testicular Germ Cell Tumors (TCGA, PanCancer Atlas)
Thymoma (TCGA, PanCancer Atlas)
Thyroid Carcinoma (TCGA, PanCancer Atlas)
Uterine Carcinosarcoma (TCGA, PanCancer Atlas)
Uterine Corpus Endometrial Carcinoma (TCGA, PanCancer Atlas)
Uveal Melanoma (TCGA, PanCancer Atlas)

Figure S1: Summary of the TGF-B Pathway, its Role in Cardiac Fibrosis/TNBC and Pharmacological Targets

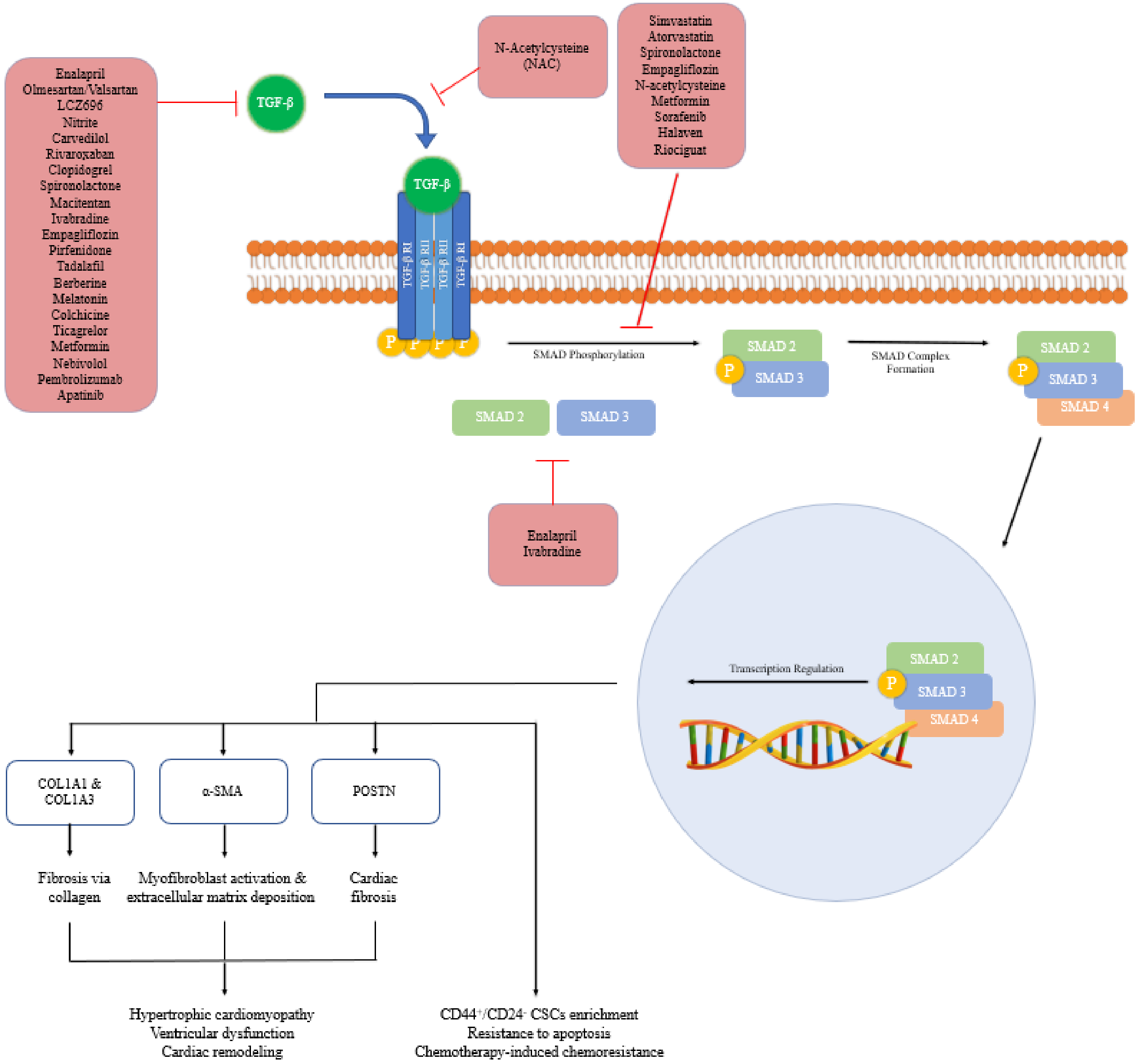

Supplement: Supplementary file 1 [file cancers-14-01577-s001.zip › cancers-1603690-supplementary.pdf]
